# NBI - plasma vaporization hybrid approach in bladder cancer endoscopic management

**Published:** 2014-06-25

**Authors:** F Stănescu, B Geavlete, D Georgescu, M Jecu, C Moldoveanu, L Adou, C Bulai, C Ene, P Geavlete

**Affiliations:** "Saint John" Emergency Clinical Hospital, Department of Urology

**Keywords:** non-muscle invasive bladder tumors, narrow band imaging (NBI) cystoscopy, bipolar plasma vaporisation

## Abstract

Abstract

Objectives: A prospective study was performed aiming to evaluate the surgical efficacy, perioperative safety profile, diagnostic accuracy and medium term results of a multi-modal approach consisting in narrow band imaging (NBI) cystoscopy and bipolar plasma vaporization (BPV) when compared to the standard protocol represented by white light cystoscopy (WLC) and transurethral resection of bladder tumors (TURBT).

Materials & Methods: A total of 260 patients with apparently at least one bladder tumor over 3 cm were included in the trial. In the first group, 130 patients underwent conventional and NBI cystoscopy followed by BPV, while in a similar number of cases of the second arm, classical WLC and TURBT were applied. In all non-muscle invasive bladder tumors’ (NMIBT) pathologically confirmed cases, standard monopolar Re-TUR was performed at 4-6 weeks after the initial intervention, followed by one year’ BCG immunotherapy. The follow-up protocol included abdominal ultrasound, urinary cytology and WLC, performed every 3 months for a period of 2 years.

Results: The obturator nerve stimulation, bladder wall perforation, mean hemoglobin level drop, postoperative bleeding, catheterization period and hospital stay were significantly reduced for the plasma vaporization technique by comparison to conventional resection. Concerning tumoral detection, the present data confirmed the NBI superiority when compared to standard WLC regardless of tumor stage (95.3% vs. 65.1% for CIS, 93.3% vs. 82.2% for pTa, 97.4% vs. 94% for pT1, 95% vs. 84.2% overall). During standard Re-TUR the overall (6.3% versus 17.4%) and primary site (3.6% versus 12.8%) residual tumors’ rates were significantly lower for the NBI-BPV group. The 1 (7.2% versus 18.3%) and 2 (11.5% versus 25.8%) years’ recurrence rates were substantially lower for the combined approach.

Conclusions: NBI cystoscopy significantly improved diagnostic accuracy, while bipolar technology showed a higher surgical efficiency, lower morbidity and faster postoperative recovery. The combined technique offered a reduced rate of residual tumors at Re-TUR, both globally as well as for orthotopic tumors. Substantially lower recurrence rates were found at 1 and 2 years among the NBI-BPV cases.

## Introduction

Bladder cancer is the most common malignancy of the urinary tract. The incidence of this pathology in the European Union is 27 to 100000 for men and 6 to 100000 for women [**1**]. 

 Standard white light cystoscopy (WLC) displayed substantial limitations as standard diagnostic modality in bladder cancer assessment due to insufficient visual accuracy, improper delimitation of flat lesions and a significant proportion of tumors being left behind [**2**]. Therefore, narrow band imaging (NBI) cystoscopy was developed as a complementary method aimed to improve tumor detection based on an optical technology advance [**3**]. Basically, it is related to the white light being filtered into 2 discrete centre wavelengths for blue (415 nm) and green (540 nm). As a consequence, the capillary vessels appear dark brown and the veins appear green, thus contrasting with the white-pink normal mucosa’ background [**4**]. 

 The current treatment of non-muscle invasive bladder tumors (NMIBT) is commonly represented by monopolar transurethral resection (TURBT). However, during the last years, the introduction of bipolar electrosurgery brought significant improvements in NMIBT therapeutic management in light of the substantially reduced bleeding risks, perioperative complications and postoperative recovery [**5**]. 

 Furthermore, the bipolar plasma vaporization of bladder tumors (BPV-BT) combined with biopsy bipolar resection was introduced as a promising alternative of minimizing surgical morbidity and convalescence periods while dealing with large volume’ tumor formations [**6**]. 

 Based on these premises, the bladder cancer diagnostic and treatment approach found a way towards substantial amelioration due to the more precise tumor detection and superior tumor ablation, as a combined NBI-BPV technique began to gain acknowledgement as a feasible alternative [**7**].

## Materials and Methods

A prospective study was performed aiming to evaluate the medium term results of a multi-modal approach consisting in NBI cystoscopy and bipolar surgery in cases of large non-muscle invasive bladder tumors. The trial evaluated the diagnostic accuracy, perioperative and follow-up results of this new diagnostic and treatment alternative, with a particular interest in the NMIBT 2 year’ recurrence rates. A comparison to the standard protocol consisting of classical cystoscopy and monopolar TURBT was concomitantly achieved. 

 A total of 260 patients with apparently at least one bladder tumor over 3 cm were included in the trial based on abdominal ultrasound, contrast CT and standard cystoscopy. The cases were equally divided in two groups while all the procedures were successfully carried out under spinal anesthesia by experienced endourologists. 

**Fig. 1:**
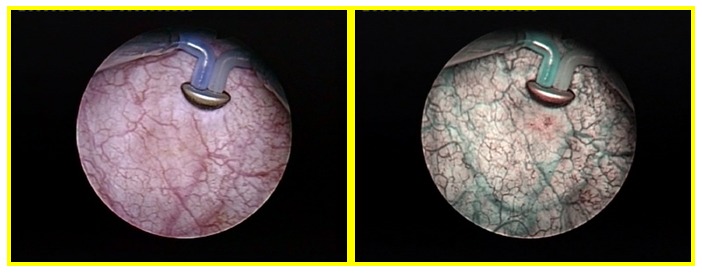
Standard and NBI cystoscopic image

 In the first group, 130 patients underwent standard and NBI cystoscopy resulting in separate bladder maps of lesions detected during either type of investigation (**Fig. 1**). As far as the actual tumor ablation was concerned, lesions visible during conventional cystoscopy were removed in white light while tumors only detected in NBI were resected using this optical enhancement mode.

**Fig. 2:**
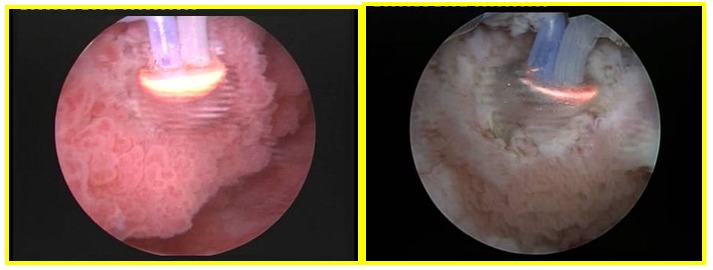
The bipolar plasma vaporization of the large tumoral bulk

 Concerning the BPV technique, tumor biopsy was initially performed with a single wire resection loop, followed by the main operating time, respectively the plasma vaporization followed by bipolar resection of the tumoral bed area and margins. The BPV-BT procedure (**Fig. 2**) was performed using the "button" type hemispherical shape electrode (Olympus Europe, Hamburg, Germany). At the end of the intervention, NBI cystoscopic control was applied for assessing the margins of the resection areas as well as for identifying eventual residual lesions. 

 In the second arm, 130 patients underwent classical WLC and TURBT.

A single postoperative chemotherapic instillation with doxorubicin or epirubicin was applied during the first 24 hours after surgery. In light of the pathological outcomes, all patients in the two groups confirmed with muscle-invasive bladder cancer were excluded from the study. In all NMIBT cases, standard monopolar Re-TUR was performed at 4-6 weeks after the initial intervention, followed by one year’ BCG immunotherapy. The follow-up protocol included abdominal ultrasound, urinary cytology and WLC, performed every 3 months for a period of 2 years. 

## Results

The average patients’ age was 65.2 years (range 32-87 years) for the combined approach’ (NBI and bipolar technology) group and 64.8 years (range 33-86 years) for the standard protocol’ series. All procedures were successfully performed in both study arms. The muscle invasive bladder cancer cases (18 in the NBI-BPV and 21 in WLC-TURBT series) were excluded from the study. 

 Regarding the perioperative parameters, the obturator nerve stimulation, bladder wall perforation, mean hemoglobin level drop and postoperative bleeding rates were significantly reduced for plasma vaporization by comparison to conventional resection (**Table 1**). The NBI-BPV patients benefited from a shorted convalescence period, as shown by the substantially decreased catheterization period (47.8 versus 74.6 hours) and hospital stay (2.8 days versus 4.2 days).

**Table 1:** Perioperative parameters

Perioperative parameters	NBI-BPV 112	TURBT-WLC 109
Obturator nerve stimulation	3 (2.7%)	20 (18.4%)
Bladder wall perforation	1 (0.9%)	7 (6.4%)
Mean hemoglobin drop	0.4 g/dl	0.9 g/dl
Postoperative bleeding	6 (5.4 %)	9 (8.3%)
Blood transfusion	1 (0.9%)	4 (3.7%)
Catheterization period	47.8 hours	74.6 hours
Hospital stay	2.8 days	4.2 days

 Concerning tumoral detection, the present data confirmed the NBI superiority when compared to standard WLC regardless of tumor stage (**Table 2**). Additionally, pathologically confirmed positive tumoral margins only discovered and resected when using NBI were discovered in 9.8 % of cases. 

**Table 2:** Tumors’ detection rates

Tumors’ detection rates	WLC	NBI cystoscopy
CIS	28 (65.1%)	41 (95.3%)
pTa	134 (82.2%)	152 (93.3%)
pT1	109 (94%)	113 (97.4%)
Overall	271 (84.2%)	306 (95%)

 During the standard Re-TUR applied at 4-6 weeks after the initial operation, the overall (6.3% versus 17.4%) and primary site (3.6% versus 12.8%) residual tumors’ rates were significantly lower for the NBI-BPV group. 

**Table 3:** Re-TUR rates

Re-TUR rates	NBI-BPV 112	WLC-TURBT 109
Overall residual tumors’ rate	7 (6.3%)	19 (17.4 %)
Primary site recurrences	4 (3.6%)	14 (12.8%)

 A total of 97 and respectively 93 patients of the NBI-BPV and WLC-TURBT series completed the 2 year’ follow-up protocol and were subsequently taken into consideration when evaluating the long term recurrences. The 1 (7.2% versus 18.3%) and 2 (11.5% versus 25.8%) year’ recurrence rates were substantially lower in the NBI-BPV group (**Table 3**). 

**Table 4:** Recurrence rates

Recurrence rates	NBI-BPV 97	WLC-TURBT 93
1 year	7.2%	18.3%
2 year	11.5%	25.8%

## Discussion

In this article, there were evaluated two new endoscopic methods and compared with the standard protocol. The main objective was to demonstrate that the respective combined approach is superior to the conventional treatment while aiming to achieve a correct tumor staging and a complete ablation of all existing lesions. 

 NBI optical technology increases the visibility of capillaries and other surface structures, the white light being filtered into 2 discrete centre wavelengths for blue (415 nm) and green (540 nm) [**8**]. Consequently, a more precise delineation of urothelial lesions and a superior contrast from normal mucosa are achieved due to the excellent imaging of the specific tumoral vascular architecture [**8**]. 

 When compared to the classical WLC, the present results concerning NBI detection rate was in accordance with the published trials [**9**], (95% vs. 84.2% in our series, 94.7% vs. 79.2% according to the literature data, respectively). 

 In respect of certain aspects of tumor pathology, the available articles support the idea that CIS patients particularly benefited from NBI technology, as it provided a substantially higher detection rate by comparison to the standard assessment [**10**]. In the respective study, the CIS detection rate was substantially improved for NBI imaging by comparison to the standard approach, in a similar manner to the present analysis (95.3% versus 65,1%) . 

 Regarding our study protocol, a special indication for NBI evaluation was constituted by large bladder tumors, frequently associated with extensive tumor margins or small satellite/ concomitant tumors, both hardly noticeable during standard cystoscopy [**11**]. 

 Bipolar technology in general and plasma vaporization in particular emphasized reduced complication rates when drawing a parallel to monopolar resection. 

 In this comparative trial, BPV provided satisfactory obturator nerve stimulation (2.7% versus 18.4%) and bladder wall perforation (0.9% versus 6.4%) rates as well as reduced mean hemoglobin drop (0.4 g/dl versus 0.9 g/dl), catheterization period (47.8 hours versus 74.6 hours) and hospital stay (2.8 days versus 4.2 days). 

 The bladder wall perforation risk, one of the most common adverse events encountered during monopolar TURBT (6.5% according to literature [**12**]) was significantly decreased in the bipolar electrosurgery setting (0.9% vs.6.4%). 

 After a 5 year’ experience, it can be stated that the bipolar plasma vaporization technique using the Olympus "button-type" special electrode is able to provide the conditions for a fast, safe and efficient malignant tissue ablation phenomenon to be achieved, particularly useful in large tumor formations [**11**]. 

 Concerning the operation time, an accurate comparative analysis cannot be outlined due to the additional NBI cystoscopic evaluation and control at the end of surgery, which, although technically simple, remains time consuming. However, according to the previously reported experience, it can be stated that the bipolar approach’ operation time is comparable with the one specific for monopolar resection [**13**]. 

 Bipolar plasma vaporization gradually disposed of the tumoral tissue, layer by layer, from the surface and progressing down to the tumor base (the "hovering" type technique), thus allowing the removal of large size lesions with improved visualization due to reduced blood loss [**11**]. 

 The use of bipolar resection for a correct pathological diagnosis of bladder tumors is a very important part of the BPV-BT protocol. The literature data showed that tumoral tissue obtained by bipolar resection displays a good quality as far as the obtained specimens are concerned [**14**]. 

 Also, another advantage of the vaporization electrode primarily refers to cases of tumors located in difficult parts of the bladder and therefore harder to resect by loop electrocautery (dome and anterior wall). In such situations, the lesions in question seem to be accessed rather easily with minimal blood loss and no bladder perforations [**15**]. 

 At Re-TUR, the residual tumors rate was substantially reduced in the NBI – bipolar approach group (6.3% versus 17.4%), while the residual tumors’ rate in the conventional protocol group was similar to the literature data (17.4% versus 25%) [**16**]. The respective progress was the consequence of a significantly decreased orthotopic recurrence rate by comparison to the control group of this study (3.6% versus 12.8%). 

 Intuitively speaking, this quite important improvement was mostly related to a better local control during the tumor ablation process obtained due to the use of the plasma vaporization phenomenon. Secondarily, the finding at the NBI control performed at the end of surgery of additional positive tumoral margins of otherwise apparently completely resected tumors in white light could provide additional explanation for this improvement. 

 In the present trial, the recurrence rates at 1 and 2 years for the WLC-TURBT group were also similar with the published data (18,3% versus 18% at 1 year; 25.8% versus 29% at 2 years) [**17**]. On the other hand, a substantially superior oncological outcome was outlined among the NBI-BPV patients (7.2% at 1 year, 11.5 % at 2 years), thus suggesting an optimistic perspective as to the favorable impact of this hybrid technique.

## Conclusions

NBI cystoscopy significantly improved diagnostic accuracy when facing a comparison to the standard WLC, as substantially more NMIBT cases as well as lesions were found during a prospective analysis. From another perspective, when compared to monopolar TURBT, the bipolar technology, primarily due to the bipolar plasma vaporization process, showed a significantly higher surgical efficiency, decreased perioperative morbidity and faster postoperative recovery in cases of large non-muscle invasive bladder tumors. 

 The combined NBI-BPV technique offered a significantly reduced Re-TUR residual tumors’ rate, both globally and for orthotopic recurrences in particular. The substantially lower medium term recurrence rates discovered at 1 and 2 years completed the superior oncological profile of this procedure. 

 In any case, further studies, more extensive series of patients and longer evaluation periods will be required in order to establish the true place of NBI cistoscopy and plasma-button vaporization in NMIBT management.
